# Influence of extreme temperatures on out-of-hospital cardiac arrest cases in Hungary: a national time-series analysis

**DOI:** 10.1016/j.resplu.2025.101194

**Published:** 2025-12-17

**Authors:** Bettina Nagy, Ádám Pál-Jakab, Boldizsár Kiss, Anna Morvai, Bence Sipos, György Pápai, Gábor Csató, Gábor Orbán, Nora Boussoussou, Béla Merkely, Péter Sótonyi, András Gerencsér, Brigitta Szilágyi, Endre Zima

**Affiliations:** a Heart and Vascular Centre, Semmelweis University, 1122 Budapest, Hungary; b Hungarian National Ambulance Service (HNAS), Markó Street 22, 1055 Budapest, Hungary; c Budapest University of Technology and Economics, Műegyetem rkp. 3, 1111 Budapest, Hungary; d Corvinus University of Budapest, Fővám tér 8, 1093 Budapest, Hungary; e Anaesthesiology and Perioperative Care Institute, Semmelweis University, Hungary; f Pannon University, Faculty of Engineering, Egyetem Street 10, 8200 Veszprém, Hungary

**Keywords:** Out-of-hospital cardiac arrest (OHCA), cardiac arrest (CA), Cardiopulmonary resuscitation (CPR), Temperature, Extreme heat, Extreme cold, Meteorological factors, sudden cardiac arrest (SCA)

## Abstract

**Introduction:**

Out-of-hospital cardiac arrest (OHCA) represents a critical public health challenge, with poor survival. Our objective was to evaluate the association between extreme temperature events and OHCA incidence in Hungary, and to assess the potential influence of additional meteorological factors, including humidity and solar radiation.

**Methods:**

We conducted a national time-series analysis of 116,579 adult OHCA cases from November 1, 2018, to December 31, 2023. Using negative binomial regression with cluster-robust standard errors, we estimated associations between daily OHCA counts and extreme temperature events, controlling for day-of-week, seasonality, and long-term trends. “Added-effect” models isolated risk attributable to sustained events while controlling for underlying non-linear temperature-health relationships through natural cubic splines. Distributed lag non-linear models (DLNM) characterized exposure-lag-response patterns over 21 days.

**Results:**

The temperature-OHCA relationship exhibited a characteristic U-shape with minimum risk at 19.0 °C. Sustained cold spells (≥2 days with daily minimum temperature ≤−9.2 °C, 2nd percentile) were associated with the highest risk increase (IRR 1.189; 95 % CI: 1.089–1.299; *p* < 0.001). Sustained heatwaves (≥3 days with daily average temperature ≥27.1 °C, 95th percentile) also significantly increased risk (IRR 1.110; 95 % CI: 1.032–1.195; *p* = 0.005). Single severe cold days (minimum temperature <−10 °C) carried an IRR of 1.143 (95 % CI: 1.012–1.291; *p* = 0.031). DLNM analysis revealed distinct temporal patterns: heat effects were acute and transient (peak at days 2–4, resolved by day 7), while cold effects were delayed and persistent (emerging at day 3, sustained beyond 14 days).

**Conclusion:**

Prolonged extreme temperatures represent independent cardiovascular hazards beyond isolated daily exposures. The immediate impact of heat and the delayed, persistent effect of cold carry important implications for public health preparedness, emergency service planning, and the timing of clinical advisories.

## Introduction

Sudden cardiac arrest (SCA), including both out-of-hospital (OHCA) and in-hospital cardiac arrest (IHCA), represents one of the most severe and life-threatening manifestations of cardiovascular disease and remains a leading cause of cardiovascular mortality.^1^ The incidence of SCA is typically estimated based on studies conducted in developed countries. OHCA is recorded in approximately 70 % of European countries, but unfortunately, data collection is not uniform. In collaboration with the EUropean REgistry of Cardiac Arrest, data from 28 European countries were analyzed, the incidence of the Emergency Medical Services (EMS) treated OHCA showed wide variability, ranging from 1.7 to 24.6 per 100,000 population per year, with a population-weighted mean of 4.0 per 100,000 per year.^2^ The incidence is 3–4 times higher in males than in females and increases with the aging of the population.^3^

Meteorological factors are getting more in the focus of research as important determinants of SCA incidence.^4^, ^5^, ^6^, ^7^, ^8^ Heatwaves impair thermoregulation and vasoreactivity, especially when accompanied by high humidity, while cold exposure increases vascular resistance and blood pressure, elevating myocardial workload. Weather changes and air pollution can further exacerbate cardiovascular vulnerability.^9^, ^10^, ^11^, ^12^, ^13^ High humidity reduces the effectiveness of sweating, leading to greater heat stress, whereas lower humidity facilitates heat dissipation and may reduce risk. In the elderly, even short periods of dehydration can impair alertness and concentration.^14^ Cold weather additionally contributes to arrhythmia triggers and interacts with air pollution, further increasing risk, particularly in older patients with pre-existing heart disease.^15^, ^16^

Most previous research on weather-related cardiac arrest has focused on Western and Southern Europe, while current European heat-health action plans are largely based on data from these regions.^17^ Central European countries may display distinct vulnerability profiles due to climatic, infrastructural, socio-economical and socio-behavioral factors. As extreme weather events become more frequent and severe, region-specific data-analysis based evidence is essential to support public health interventions. Understanding local weather-health relationships is also critical for health system resource planning, the development of early warning systems, and meteorological data based national emergency preparedness.^18^

This research aimed to quantify the independent associations between sustained extreme temperature events and OHCA incidence across Hungary, characterize the temporal patterns of these relationships using distributed lag non-linear modeling, and assess the potential influence of additional meteorological factors, including humidity and solar radiation, to provide evidence for optimizing existing weather alert systems.

## Materials and methods

### Study design and setting

We conducted a national time-series analysis using comprehensive OHCA surveillance data from the Hungarian National Ambulance Service coupled with meteorological data from the Hungarian Meteorological Service (HMS). The study period covered the time period from November 1, 2018, to December 31, 2023, including 1887 consecutive days across multiple seasonal cycles and extreme weather events.

### Data sources and case definition

#### OHCA surveillance data

The Hungarian National Ambulance Service maintains a comprehensive registry of all emergency medical service responses to OHCA events nationwide. We included all cases occurring in adults (≥18 years) during the study period.

OHCA cases were identified based on confirmed cardiac arrest requiring EMS intervention, where resuscitation attempts (cardiopulmonary resuscitation, defibrillation, and/or advanced life support) were initiated. Starting from all adult OHCA cases recorded during the study period (*N* = 147,574), exclusion criteria were applied in the predefined order (Supplementary Table S1). Cases were excluded if they involved traumatic etiology, suicide attempts, substance-related arrests, obstetric emergencies, or drowning incidents, focusing the analysis on medical cardiac arrests potentially influenced by ambient temperature. OHCA events with incomplete data (primarily missing geographic coordinates or unsuccessful meteorological linkage) were also excluded.

Cases in which EMS was dispatched solely for death examination without attempted resuscitation were likewise excluded. This approach ensured that the analytic cohort reflected EMS-attended, temperature-related medical cardiac arrests and reduced the risk of misclassification, as unattended deaths, particularly during cold periods which may reflect delayed discovery rather than acute cardiac events. The final analytic dataset comprised 116,579 EMS-attended medical OHCA cases.

#### Meteorological data

Daily weather data were obtained from the HMS representative weather station network, providing complete coverage of temperature, humidity, and solar radiation measurements across Hungary. Data completeness exceeded 99.5 % for all temperature variables throughout the study period.

#### Exposure definitions

Primary extreme temperature exposures were defined using season- and region-specific percentile thresholds to reflect local climatic adaptation:

• **Primary heatwave**: ≥3 consecutive days with a daily mean temperature of ≥27.1 °C, corresponding to the 95th percentile of warm-season (May–September) temperatures.
• **Primary cold spell**: ≥2 consecutive days with a daily minimum temperature of ≤−9.2 °C, corresponding to the 2nd percentile of cold-season (November–March) temperatures.
• **Severe cold day**: any single day with a minimum temperature below −10 °C.

Percentile thresholds were calculated separately for the warm and cold seasons to ensure climatological relevance. In addition to these primary definitions, we also examined secondary exposures**:**

• Hot day: daily maximum temperature ≥35 °C.
• Tropical night: daily minimum temperature ≥20 °C.
• Alternative heatwaves: ≥2 consecutive days with mean temperature ≥27.1 °C (95th percentile), ≥2 consecutive days ≥28.2 °C (98th percentile), or ≥2 consecutive days ≥28.8 °C (99th percentile).
• Alternative cold spells: ≥2 consecutive days with minimum temperature ≤−6.0 °C (5th percentile), ≤-12.1 °C (1st percentile), or ≥3 consecutive days with minimum temperature ≤−6.0 °C (5th percentile).
• Low solar radiation: ≤109.0 J/cm^2^ (25th percentile).
• High solar radiation: ≥281.4 J/cm^2^ (75th percentile).
• Low humidity: <50 %.
• High humidity: >70 %.

The climatological distributions and exposure frequencies for these definitions are presented in Supplementary Figs. S1 and S2.

### Statistical analysis

#### Primary modeling approach

Since daily OHCA counts exhibited substantial variability beyond what would be expected under a Poisson distribution (Pearson *χ*^2^ = 1.70), we applied negative binomial regression as the primary analytical method. This approach is well suited for overdispersed count data, providing more reliable estimates of variance and effect size. To minimize confounding from temporal factors, the models included fixed effects for day of the week, calendar month, and study year, thereby accounting for weekly patterns, seasonal variation, and long-term trends. In addition, cluster-robust standard errors were applied at the year-month level to correct for residual autocorrelation not fully captured by these fixed effects. For categorical exploratory analyses, daily temperature was initially represented using categories such as frost, ice, and severe cold days, with non-extreme days serving as the reference. However, the primary models focused on sustained extreme event definitions, including heatwaves, primary cold spells, and severe cold days, which are reported in the main results. The full model specification, including the formal equation, is presented in the Supplementary Methods.

#### Added-effect model specification

To isolate the risk attributable to sustained extreme temperature events beyond the general temperature-risk curve, we applied an “added-effect” model. This approach incorporated binary indicators for heatwaves and cold spells while simultaneously adjusting for the underlying non-linear association between daily temperature and OHCA risk using natural cubic splines. The full model specification is provided in the Supplementary Methods.

#### Distributed lag non-linear modeling

We used DLNM to examine how temperature affects OHCA risk over time, allowing us to capture both immediate and delayed effects. This approach makes it possible to model the complex, non-linear relationship between temperature and health outcomes while also accounting for lagged effects that may occur days or even weeks after exposure. To ensure physiological plausibility, the model was centered at the empirically derived minimum mortality temperature (19 °C) and evaluated across a 21-day lag window, which has been shown to capture both the short-term impact of heat and the more delayed effects of cold. Full technical details of the cross-basis function and spline specifications are provided in the Supplementary Methods.

Sex was classified based on EMS records. Sex-specific analyses were conducted by fitting separate negative binomial models for men and women using the same model specification as the primary analysis. We additionally tested for effect modification by including a sex × exposure interaction term.

### Sensitivity analyses

Robustness was assessed through multiple approaches: (1) Poisson versus negative binomial model comparison, (2) leave-one-season-out cross-validation, (3) alternative clustering schemes, (4) sex-stratified analysis with formal interaction testing, and (5) seasonal adjustment sensitivity analysis.

### Statistical software

Analyses were conducted using Python 3.8.20 (pandas, statsmodels) for data processing and primary modeling, and R 4.1+ (dlnm, MASS, sandwich) for DLNM analysis. Statistical significance was set at *α* = 0.05.

### Ethical approval and trial registration

This study was conducted in strict accordance with the ethical guidelines and regulations set forth by the National Ethics Committee of Hungary. Ethical approval was granted for the collection and analysis of data related to OHCA cases, integral to our observational assessment of OHCA epidemiology, regional variations, and mortality rates in Hungary. The study was assigned ethical approval numbers (Reference Numbers: IV/3043/2021/EKU and IV/3043-3/2021/EKU). All procedures and data handling adhered strictly to established ethical standards and regulations and were conducted in accordance with the Declaration of Helsinki.^19^

## Results

### Study population and exposure characteristics

During 1887 study days, 116,579 adult OHCA cases were included. Median daily incidence was 60 (52–69). Mean age was 69.4 ± 14.3 years and 59 % of patients were male. Most arrests occurred at home (80.5 %). Daily mean temperature ranged from −6.8 °C to 30.6 °C. Extreme events were infrequent, with 20 heatwave days, 14 cold spell days, and 12 severe cold days. The climatological distribution of warm- and cold-season temperatures and the frequency of exposure days for alternative heatwave and cold spell definitions are presented in Supplementary Figs. S1 and S2. Detailed baseline and exposure characteristics are presented in Table 1.

**Table 1:** Baseline epidemiological and meteorological characteristics of the Hungarian OHCA cohort and study period. Values are shown for the complete 5.2-year dataset. **Abbreviations:** IQR, interquartile range; OHCA, out-of-hospital cardiac arrest; SD, standard deviation.

**Characteristic**	**Value**
**Study period and analytical scope**	
Total analysis days, *n*	1887
Study duration	5.2 years
Date range	November 1, 2018–December 31, 2023

**Out-of-hospital cardiac arrest cases**	
Total OHCA cases, *n*	116,579
Daily OHCA count, median (IQR)	60.0 (52.0–69.0)
Daily OHCA count, range	28–102

**Patient demographics**	
Mean age ± SD, years	69.4 ± 14.3
Male patients, *n* (%)	68,735 (59.0)
Female patients, *n* (%)	47,844 (41.0)

**Location of OHCA**	
Home, *n* (%)	93,808 (80.5)
Public areas, *n* (%)	12,271 (10.5)
Other locations, *n* (%)	10,500 (9.0)

**Meteorological exposure summary**	
Daily mean temperature range, °C	−6.8 °C to 30.6 °C
Primary heatwave days, *n* (%)	20 (1.1)
Primary cold spell days, *n* (%)	14 (0.7)
Individual severe cold days, *n* (%)	12 (0.6)

**Temperature thresholds**	
Heatwave threshold (95th percentile), °C	27.1
Cold spell threshold (2nd percentile), °C	−9.2
Severe cold threshold, °C	−10.0

### Overall temperature-OHCA relationship

**Fig. 1:**
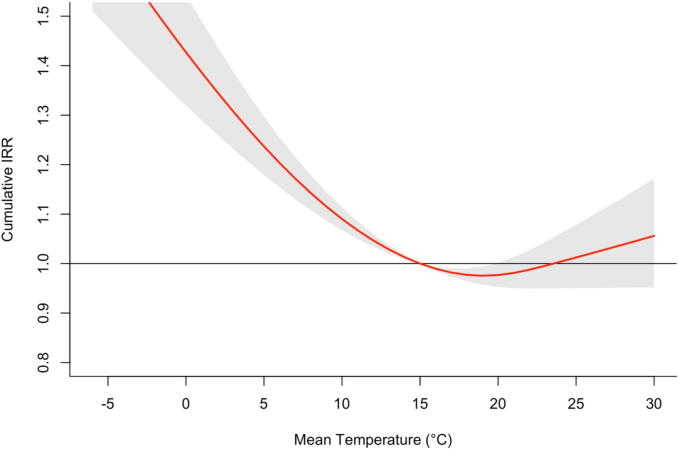
**Overall cumulative exposure-response**. Cumulative association between daily mean temperature and OHCA risk over a 21-day lag, estimated with distributed lag non-linear models. The curve shows a U-shaped relationship with lowest risk at 19.0 °C (minimum mortality temperature). Risk increases towards both heat and cold extremes, with steeper effects for cold. The red line indicates the estimated cumulative IRR; gray shading indicates 95 % confidence intervals. **Abbreviations:** CI, confidence interval; IRR, incidence rate ratio; MMT, minimum mortality temperature; OHCA, out-of-hospital cardiac arrest.

The DLNM analysis revealed a non-linear, U-shaped association between ambient temperature and cumulative OHCA incidence over 21 days (Fig. 1). The minimum mortality temperature was identified at 19.0 °C. Risk increased significantly at both temperature extremes, with a steeper gradient observed for cold compared to heat exposures.

### Temporal patterns of temperature effects

**Fig. 2:**
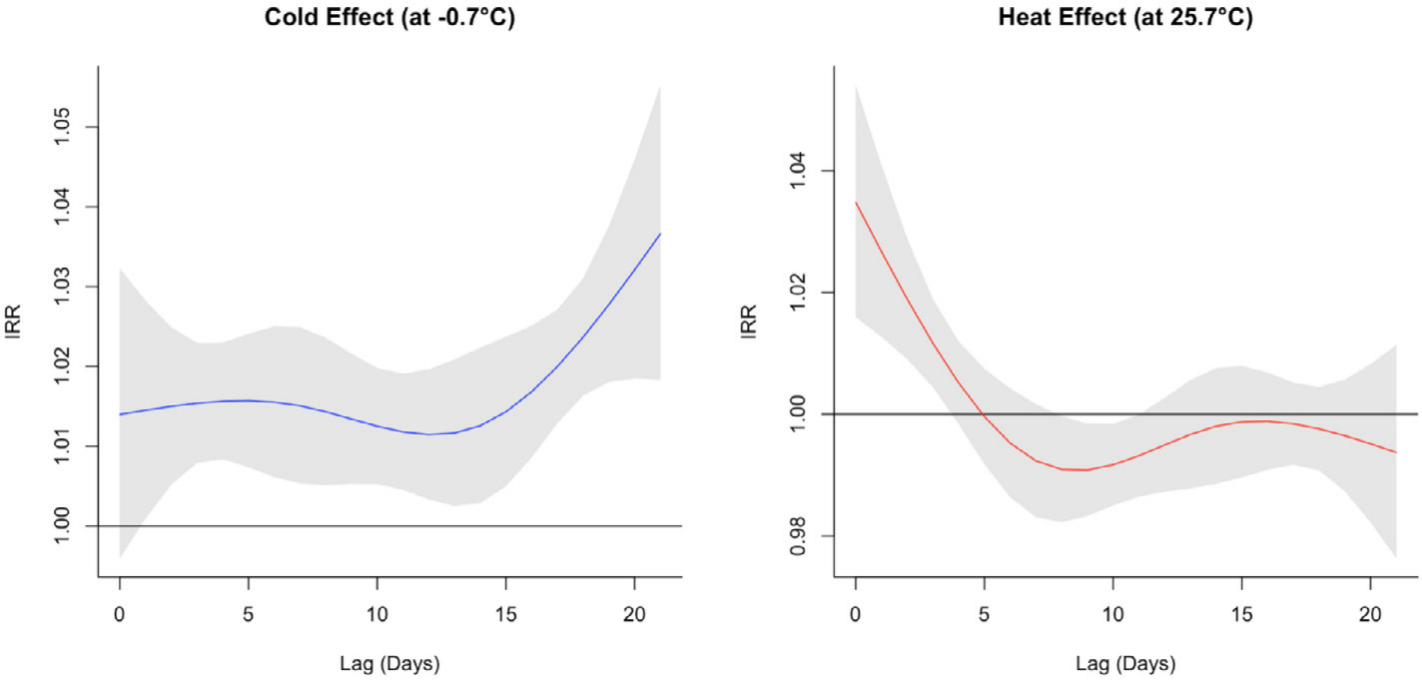
**Lag-specific effects of cold and heat exposure on OHCA incidence**. Temporal patterns of OHCA risk associated with cold (left) and heat (right) exposures over a 21-day lag, estimated with distributed lag non-linear models. Curves are shown relative to the minimum mortality temperature (19.0 °C, IRR = 1.0). Heat (95th percentile, 25.7 °C) produced an acute, short-lived increase in risk, whereas cold (5th percentile, −0.7 °C) produced a delayed and more persistent elevation in OHCA case incidence. Solid lines represent mean estimates; shaded areas show 95 % confidence intervals. **Abbreviations:** CI, confidence interval; IRR, incidence rate ratio; OHCA, out-of-hospital cardiac arrest. The 95th (25.7 °C) and 5th (−0.7 °C) percentiles shown here reflect the daily temperature distribution used in the DLNM framework to illustrate lag-specific effects, and therefore differ from the sustained extreme event thresholds applied in the primary analyses.

The DLNM showed distinct lag patterns for heat and cold effects as shown in Fig. 2. Heat at the 95th percentile (25.7 °C) was associated with an acute increase in OHCA risk, peaking within the first 4 days and resolving by day 7. In contrast, cold at the 5th percentile (−0.7 °C) was associated with a delayed response, emerging around day 3 and remaining elevated throughout the 21-day lag period.

### Primary findings: sustained extreme weather events

Using added-effect models, all three primary exposures were significantly associated with higher OHCA incidence detailed in Table 2. Cold spells showed the strongest effect (IRR 1.189, 95 % CI 1.089–1.299), followed by severe cold days (IRR 1.143, 95 % CI 1.012–1.291) and sustained heatwaves (IRR 1.110, 95 % CI 1.032–1.195).

**Table 2:** Independent effects of extreme temperature exposures on daily OHCA incidence, estimated with added-effect models. Heatwaves were defined as ≥3 consecutive days with mean temperature ≥27.1 °C (95th percentile, warm season), cold spells as ≥2 consecutive days with minimum temperature ≤−9.2 °C (2nd percentile, cold season), and severe cold days as single days with minimum temperature below −10 °C. **Abbreviations:** CI, confidence interval; df, degrees of freedom; IRR, incidence rate ratio; *T*_avg_, daily average temperature; *T*_min_, daily minimum temperature; OHCA, out-of-hospital cardiac arrest.

**Exposure category**	**Definition**	**IRR (95 % CI)**	***p*-value**
Extreme heat	Sustained heatwave: ≥3 consecutive days with *T*_avg_ ≥ 27.1 °C (95th percentile)	1.110 (1.032–1.195)	0.005
Extreme cold	Cold spell: ≥2 consecutive days with *T*_min_ ≤ −9.2 °C (2nd percentile)	1.189 (1.089–1.299)	<0.001
Extreme cold	Severe cold day: Single day with *T*_min_ < −10 °C	1.143 (1.012–1.291)	0.031

**Fig. 3:**
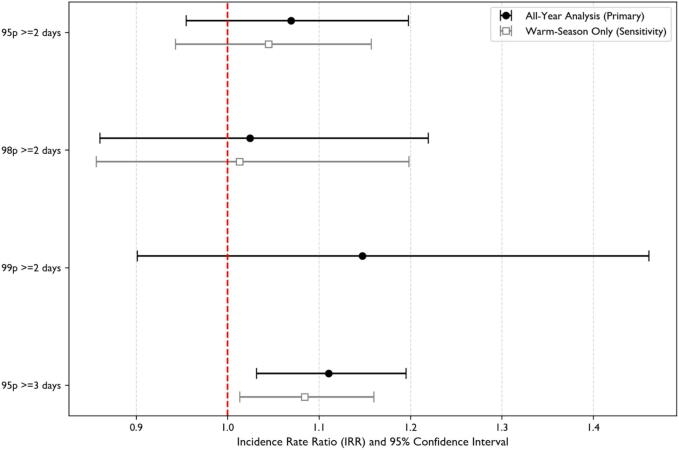
**Added-effect of heatwaves on OHCA incidence**. Each point represents the additional OHCA risk attributable to the sustained event, controlling for the underlying non-linear temperature-risk curve. Error bars denote 95 % confidence intervals; vertical line represent no effect (IRR = 1.0). **Abbreviations:** CI, confidence interval; IRR, incidence rate ratio; OHCA, out-of-hospital cardiac arrest; 95p, 95th percentile; 98p, 98th percentile; 99p, 99th percentile.

The robustness of the primary findings was confirmed across alternative exposure definitions. As shown in Fig. 3, added-effect models yielded consistent associations for different heatwave thresholds, and similar stability was observed for cold spells in the Supplementary analyses (Supplementary Fig. S3).

### Secondary weather exposures

Analyses of humidity, solar radiation, isolated hot days, and tropical nights showed no association with OHCA incidence (Supplementary Table S2; Supplementary Figs. S8 and S9). This indicates that no secondary meteorological variable demonstrated a measurable effect, and the observed associations were specific to extreme temperatures.

### Sex differences and effect modification

Formal interaction testing revealed no statistically significant differences in temperature effects between men and women (global interaction *p* = 0.226; Supplementary Table S3). Sex-stratified analyses indicated that heatwave effects were broadly similar in men and women, with no evidence of statistical interaction (Supplementary Fig. S6). For cold spells, effect estimates appeared directionally higher in women; however, confidence intervals overlapped substantially, and the female model was inherently less stable due to the limited number of cold-spell days and female cases (Supplementary Fig. S7). In line with the non-significant interaction, the primary analyses were therefore conducted in the pooled population.

### Sensitivity and robustness analyses

Primary findings remained stable across multiple sensitivity analyses (Supplementary Table S5). Negative binomial models consistently outperformed Poisson regression, with an AIC difference greater than 320. To assess whether the observed associations were driven by any single season, we performed leave-one-season-out validation, sequentially omitting each season and re-estimating the models. The cold spell effect remained statistically significant in 5 out of 6 scenarios, while the heatwave effect remained significant in 3 out of 5 scenarios (Supplementary Figs. S4 and S5).

### Validation against operational alert criteria

Analysis of official HMS weather alert thresholds provided empirical validation for existing public health protocols (Supplementary Table S4). Days meeting the criteria for HMS Heatwave Level I (≥3 days with *T*_avg_ ≥ 27.1 °C; *p* = 0.024) and Level II (≥3 days with *T*_avg_ ≥ 28.2 °C; *p* = 0.002) correspond closely to our primary and alternative sustained heatwave definitions, and were both associated with significantly increased OHCA risk. Similarly, days classified as Severe cold day alerts (*T*_min_ < −10 °C; *p* = 0.023) matched our primary severe cold day definition and showed significant risk elevation.

## Discussion

### Main findings

This nationwide analysis identified a U-shaped relationship between ambient temperature and OHCA incidence, with minimum risk at 19 °C. Heat exposure was associated with an acute and transient increase in cases, whereas cold exposure produced a delayed and prolonged effect. Both sustained events and single severe cold days were linked to higher risk of OHCA incidence.

### Comparison with literature and mechanisms

Heatwaves in our analysis were associated with an acute but short-lived rise in OHCA, with risk peaking in the first days of exposure. Similar short-term patterns have been described in Europe: a nine-city study reported that heat-related mortality was concentrated within the first 2–3 days of heatwaves,^20^ and a pan-European analysis of the exceptional summer of 2022 likewise found that excess mortality was largely confined to the initial days of extreme heat.^21^ It is important to note that overall heat-related mortality and OHCA represent distinct outcomes. While mortality captures broader population-level vulnerability, OHCA reflects sudden, acute events requiring EMS intervention. Nevertheless, both indicators demonstrate similar temporal patterns during heatwaves, with effects largely concentrated in the first days of exposure, supporting the physiological plausibility of the acute heat-related increase in OHCA.^20^, ^22^

Cold spells showed a different profile, with a higher OHCA risk emerging after several days and persisting for more than two weeks. This prolonged effect is in line with Japanese nationwide findings^23^ and with covering multicountry mortality analyses demonstrating that cold accounts for the majority of temperature-related cardiovascular deaths.^24^

We also identified an association with isolated severe cold days. Evidence from Helsinki indicates that cold days, even when not being part of longer spells, increase OHCA incidence across seasons, underscoring the acute cardiovascular stress imposed by short, intense exposures.^25^ Importantly, the association remained when the COVID-19 winter (2020/21) was excluded in sensitivity analyses, indicating that pandemic-related effects did not drive the observed cold-related increase in OHCA.

The distinct lag characteristics observed, immediate for heat and delayed for cold, are physiologically plausible. Heat stress can trigger relative and absolute hypovolaemia via vasodilation and dehydration, electrolyte imbalance, and arrhythmogenic events, whereas cold promotes vasoconstriction, blood pressure elevation, and pro-thrombotic changes, leading to a cumulative cardiovascular strain.^23^

In contrast, we did not identify a measurable independent effect of humidity on OHCA incidence. While Supplementary Fig. S9 suggests a slight upward trend at the highest humidity percentiles, this pattern was not statistically significant in adjusted models, likely due to overlap with high-temperature periods and the limited number of days with extreme humidity. This interpretation remains theoretical, as environmental-physiological pathways linking humidity and OHCA are not fully understood and may be influenced by unknown confounders. Previous studies have also reported inconsistent associations between humidity and cardiovascular events, particularly when controlling for temperature.^20^

### Public health and clinical implications

Our results have direct relevance for health system preparedness in continental Europe. The acute heat effect means that EMS and hospitals must anticipate sudden surges in OHCA at the onset of heatwaves, a pattern also reported in Canada^26^ and South Korea.^27^ By contrast, the prolonged cold effect requires sustained vigilance well beyond the exposure window. This is consistent with international mortality studies showing that most temperature-related deaths are attributable to cold, particularly moderate cold exposures rather than extreme frost or trauma-related events.^24^ This is consistent with international all-cause mortality studies showing that most temperature-related deaths are attributable to cold, particularly moderate cold exposures rather than extreme frost or trauma-related events.^24^ Although these findings come from mortality data, the delayed and persistent cold-related risk pattern parallels the OHCA associations observed in our analysis. Unlike many prior studies, we also demonstrated that isolated severe cold days carry excess risk, underlining the importance of flexible alert systems that capture both multi-day and single-day extremes.

This study provides novel robust data from Central Europe, where nationwide analyses of temperature-related OHCA were scarce to date. Incorporating these findings into regional warning systems, clinical practice could support more precise patient counseling, with emphasis on hydration and reduced exertion during heat, and on blood pressure management and adequate protection during cold periods. Moreover, linking weather forecasts to expected medical risks could help EMS prepare more practically, enabling staff and resources to be in place when patients are most vulnerable.

## Conclusion

This nationwide analysis demonstrates that both heatwaves and cold spells significantly increase the risk of OHCA in a Central European population, but with markedly different temporal profiles. Heat exerts an immediate and transient effect, while cold produces delayed and prolonged excess risk. These findings provide novel evidence from a continental climate and underline the need for tailored preparedness strategies: rapid response during heatwaves and extended vigilance during cold periods. Integrating temperature-related risk into public health planning, timely EMS reinforcement with HR reorganization and clinical guidance for patients are essential as climate extremes become more frequent and severe.

## Limitations of the study

Some limitations should also be acknowledged. Exposure was assigned using regional meteorological data, which may not fully capture microclimatic or indoor conditions; such non-differential misclassification would likely bias associations toward the null. Although we adjusted for major temporal confounders, data on air pollution, influenza activity, and other environmental factors were unavailable, though the short duration of extreme events makes major residual confounding less likely. The number of days meeting the most extreme definitions was limited, resulting in imprecise estimates in some subgroup analyses, particularly in sex-stratified models. A further limitation is that OHCA cases with obvious death at the scene and without attempted resuscitation were excluded, as the underlying cause of death in these situations is frequently clarified only after autopsy; therefore, these events may represent a mixed or uncertain etiology not directly comparable to EMS-treated, temperature-related cardiac arrests. Finally, while cardiovascular physiology is universal, thresholds and effect sizes observed in Hungary may not directly generalize to populations with different climates, baseline health, or healthcare systems.

## Future research directions

Future investigations should examine individual-level factors modifying weather-health relationships, explore mechanistic pathways linking solar radiation to cardiovascular events, and validate findings across different climate zones and populations. Long-term climate trend analysis with formal time-series methods could inform health system adaptation strategies for changing environmental conditions.

## CRediT authorship contribution statement

**Bettina Nagy:** Writing – original draft, Methodology, Investigation, Formal analysis, Data curation, Conceptualization. **Ádám Pál-Jakab:** Writing – review & editing, Project administration, Methodology, Data curation, Conceptualization. **Boldizsár Kiss:** Writing – review & editing, Data curation, Conceptualization. **Anna Morvai:** Writing – review & editing, Methodology, Conceptualization. **Bence Sipos:** Validation, Software, Methodology, Investigation, Data curation. **György Pápai:** Writing – review & editing, Supervision, Investigation, Funding acquisition, Conceptualization. **Gábor Csató:** Writing – review & editing, Validation, Software, Resources, Investigation, Funding acquisition, Conceptualization. **Gábor Orbán:** Writing – review & editing, Methodology, Data curation. **Nora Boussoussou:** Writing – original draft, Visualization, Validation, Software, Resources, Project administration, Methodology, Conceptualization. **Béla Merkely:** Validation, Supervision, Resources, Project administration, Data curation, Conceptualization. **Péter Sótonyi:** Writing – review & editing, Validation, Resources, Project administration, Methodology, Investigation, Conceptualization. **András Gerencsér:** Validation, Supervision, Software, Resources, Project administration, Methodology, Conceptualization. **Brigitta Szilágyi:** Writing – review & editing, Validation, Methodology, Formal analysis, Data curation. **Endre Zima:** Writing – review & editing, Validation, Supervision, Resources, Project administration, Methodology, Investigation, Formal analysis, Data curation, Conceptualization.

## Funding

Project no. TKP2021-EGA-02 has been implemented with the support provided by the Ministry of Culture and Innovation of Hungary from the National Research, Development and Innovation Fund, financed under the TKP2021-EGA funding scheme.

Semmelweis University provided open-access publication funding. Research support was received through the PhD Excellence Program of Semmelweis University via grant EFOP-3.6.3-VEKOP-16-2017-00009 (“Semmelweis 250+ Excellence Scholarship”). Project no. RRF-2.3.1-21-2022-00014 received implementation support from the European Union through the Hungarian Climate Change National Laboratory. Additional support was provided by RRF-2.3.1-21-2022-00003 under the National Cardiovascular Laboratory Artificial Intelligence Core Lab framework.

This research was supported by the EKÖP-2024-138 New National Excellence Program, funded by the Ministry for Culture and Innovation through the National Research, Development and Innovation Fund.

E.Z.’s disclosures include direct personal payments (speaker fees, study-related support) from Abbott, AstraZeneca, Biotronik, Boston Scientific, Innomed, and Medtronic, as well as former roles as National Representative of ACVCA on behalf of the Hungarian Society of Cardiology and past president of its Working Group on Cardiac Pacing and Arrhythmias.

B.M. has received direct personal payments (speaker fees, study-related support) from Boehringer Ingelheim, Daiichi Sankyo, DUKE Clinical Institute, and Novartis, and institutional grants to the Heart and Vascular Center of Semmelweis University from Biotronik, Boehringer Ingelheim, DUKE Clinical Institute, Eli Lilly, and Novartis.

## Declaration of competing interest

B.M. reports grants from Boston Scientific, Medtronic and personal fees from Biotronik, Abbott, AstraZeneca, Boehringer Ingelheim, Novartis, outside the submitted work. L.G. reports grants from Medtronic and personal fees from Biotronik, Abbott, AstraZeneca, Boehringer Ingelheim, outside the submitted work. E.Z. reports lecture and advisory fees outside the submitted work from Biotronik, Medtronic, Boston Scientific, Zoll Medical, Novartis, Richter, Orion Pharma outside the submitted work.
