# Supplementary material for: Influence of extreme temperatures on out-of-hospital cardiac arrest cases in Hungary: a national time-series analysis
Source: Resusc Plus. 2025 Dec 17;27:101194. doi: 10.1016/j.resplu.2025.101194 (PMC12828408; doi:10.1016/j.resplu.2025.101194)
Supplement: Supplementary Data 1 [file mmc1.docx]

**Influence of extreme temperatures on out-of-hospital cardiac arrest cases in Hungary; a national time-series analysis**

**Supplementary Materials - Methods, Tables and Figures**

**Supplementary Methods: Page 2.**

- **Statistical Modeling** (Page 2)

**Supplementary Tables: Pages 3-9.**

- **Table S1:** Sequential Case Selection Process (Page 3)
- **Table S2:** Incidence Rate Ratios for Primary, Alternative, and Secondary Weather Exposures (Pages 4-6)
- **Table S3:** Sex-Specific Incidence Rate Ratios for Primary Heatwaves, Cold Spells, and Severe Cold Days (Page 7)
- **Table S4:** Evaluation of Hungarian Meteorological Service Weather Alert Thresholds in Relation to OHCA Incidence (Page 8)
- **Table S5:** Model Diagnostics and Sensitivity Analyses (Page 9)

**Supplementary Figures: Pages 10-17.**

- **Figure S1**: Heatwave Exposure Characteristics in the Study Period (Page 10)
- **Figure S2:** Cold Spell Exposure Characteristics in the Study Period (Page 11)
- **Figure S3:** Added Effects of Cold Spells on OHCA Incidence (Page 12)
- **Figure S4:** Stability of Primary Heatwave Finding (Leave-One-Summer-Out) (Page 13)
- **Figure S5:** Stability of Primary Cold Spell Finding (Leave-One-Winter-Out) (Page 14)
- **Figure S6:** Sex-Specific Analysis of Heatwave Effects on OHCA Incidence (Page 15)
- **Figure S7:** Sex-Specific Analysis of Cold Spell Effects on OHCA Incidence (Page 16)
- **Figure S8:** Solar Radiation Effects on Daily OHCA Incidence (Page 17)
- **Figure S9:** Humidity Effects on Daily OHCA Incidence (Page 18)

**Supplementary Methods - Statistical Modeling**

**Primary Modeling Approach**

Daily OHCA counts (Yt) were modeled using a negative binomial distribution to address significant overdispersion (Pearson χ² = 1.70). The model was specified as:

Yt ~ Negative Binomial(μt, θ)

log(μt) = β0 + β1 × TempCategoryt + γ1 × DOWt + γ2 × Montht + γ3 × Yeart

where:

μt = expected daily OHCA count

θ = dispersion parameter

TempCategoryt = categorical exposure indicator (Normal [reference], Frost, Ice, Severe Cold)

DOWt, Montht, Yeart = categorical fixed effects controlling for weekly, seasonal, and long-term trends

Cluster-robust standard errors were calculated with clustering at the year-month level to account for residual autocorrelation.

**Added-Effect Models**

To estimate the independent contribution of sustained extreme temperature events, we extended the model with a binary indicator for heatwaves or cold spells while simultaneously adjusting for the underlying non-linear temperature-risk relationship. The model took the form:

log(μt) = β0 + β1 × ExtremeEventt + f(Temperaturet) + Confounderst

Here:

f(Temperaturet) = natural cubic spline with 3 degrees of freedom applied to daily mean temperature

ExtremeEventt = binary variable (1 = heatwave or cold spell day, 0 = otherwise, as defined in Section 2.2.3)

**Distributed Lag Non-Linear Models**
Lagged associations were examined using a cross-basis function specified in the *dlnm* R package:

- Temperature dimension: natural cubic spline with knots at the 10th, 75th, and 90th percentiles of daily temperatures
- Lag dimension: natural cubic spline with 4 degrees of freedom across 21 lag days
- Centering: models were centered at the minimum mortality temperature (19.0 °C), empirically derived from the overall exposure-response curve

The 21-day lag period was chosen to reflect plausible physiological mechanisms: heat effects typically occur acutely within the first days after exposure, while cold-related cardiovascular stress often emerges with several days’ delay and persists for weeks.

**Table S1: Sequential Case Selection Process**

| **Step** | **Selection Criterion** | **N Excluded** | **N Remaining** |
| --- | --- | --- | --- |
| **1** | OHCA cases during study period (November 1, 2018 - December 31, 2023) | - | 147,574 |
| **2** | Exclude cases with missing age data | 5,182 | 142,392 |
| **3** | Exclude pediatric cases (age <18 years) | 2,610 | 139,782 |
| **4** | Exclude traumatic etiology | 1,749 | 138,033 |
| **5** | Exclude drowning | 119 | 137,914 |
| **6** | Exclude substance-related arrests | 216 | 137,698 |
| **7** | Exclude suffocation/choking | 2,176 | 135,522 |
| **8** | Exclude electrocution | 56 | 135,466 |
| **9** | Exclude incomplete data† | 18,887 | 116,579 |
|  | Final analytic cohort | - | 116,579 |

Sequential case selection from the Hungarian National Ambulance Service registry. Starting from all adult OHCA cases recorded during the study period (N=147,574), exclusion criteria were applied sequentially in the order shown. Cases meeting multiple exclusion criteria were removed at the first applicable step. The final analytic cohort comprised 116,579 cases (79.0% inclusion rate), ensuring comprehensive population coverage while maintaining focus on medical cardiac arrests with documented EMS response and resuscitation attempts.

†Incomplete data includes cases with missing or invalid geographic coordinates preventing weather exposure assignment, unsuccessful meteorological data linkage, or missing critical case identification variables.

Abbreviations: EMS, Emergency Medical Service; N: number; OHCA, out-of-hospital cardiac arrest.

**Table S2: Incidence Rate Ratios for Primary, Alternative, and Secondary Weather Exposures**

| **Weather Exposure** | **Definition** | **Exposure Days, n (%)** | **OHCA Cases, n** | **Unadjusted Rate per Day** | **Adjusted IRR (95% CI)†** | **p-value** | **FDR-Corrected p** |
| --- | --- | --- | --- | --- | --- | --- | --- |
| **PRIMARY FINDINGS** | | | | | | | |
| **Primary Heatwave** | **≥3 days, T_avg_ ≥ 27.1°C (P95)** | **20 (1.1%)** | **1,098** | **54.90** | **1.110 (1.032-1.195)** | **0.005** | **0.012** |
| **Primary Cold Spell** | **≥2 days, T_min_ ≤ -9.2°C (P02)** | **14 (0.7%)** | **981** | **70.07** | **1.189 (1.089-1.299)** | **<0.001** | **0.002** |
| **Severe Cold Day** | **T_min_ < -10°C** | **41 (2.2%)** | **2,768** | **67.51** | **1.143 (1.012-1.291)** | **0.031** | **0.058** |
| **REFERENCE CATEGORY** | | | | | | | |
| Normal Days | Temperature days not meeting extreme criteria | 1,551 (82.2%) | 94,625 | 61.01 | 1.00 (Reference) | — | — |
| **TEMPERATURE SPECTRUM** | | | | | | | |
| Frost Days | T_min_< 0°C & T_max_ ≥ 0°C | 277 (14.7%) | 18,147 | 65.51 | 0.979 (0.951-1.007) | 0.138 | 0.241 |
| Ice Days | T_max_ < 0°C & T_min_ ≥ -10°C | 47 (2.5%) | 3,039 | 64.66 | 0.985 (0.935-1.038) | 0.571 | 0.714 |
| **SECONDARY HEAT EXPOSURES** | | | | | | | |
| Hot Days | T_max_ ≥ 35°C | 40 (2.1%) | 2,144 | 53.60 | 1.034 (0.951-1.124) | 0.432 | 0.578 |
| Tropical Nights | T_min_ ≥ 20°C | 72 (3.8%) | 3,861 | 53.62 | 1.014 (0.969-1.062) | 0.540 | 0.675 |
| Heatwave Alt-1 | ≥2 days, T_avg_ ≥ 27.1°C (P95)§ | 32 (1.7%) | 1,770 | 55.31 | 1.069 (0.955-1.198) | 0.246 | 0.369 |
| Heatwave Alt-2 | ≥2 days, T_avg_ ≥ 28.2°C (P98)§ | 10 (0.5%) | 553 | 55.30 | 1.024 (0.860-1.220) | 0.786 | 0.851 |
| Heatwave Alt-3 | ≥2 days, T_avg_ ≥ 28.8°C (P99)§ | 6 (0.3%) | 370 | 61.67 | 1.147 (0.901-1.461) | 0.264 | 0.381 |
| **SECONDARY COLD EXPOSURES** | | | | | | | |
| Cold Spell Alt-1 | ≥2 days, T_min_ ≤ -6.0°C (P05)§ | 33 (1.7%) | 2,211 | 67.00 | 1.072 (0.973-1.182) | 0.160 | 0.267 |
| **Cold Spell Alt-2** | **≥2 days, T_min_ ≤ -12.1°C (P01)§** | **8 (0.4%)** | **567** | **70.88** | **1.232 (1.118-1.357)** | **<0.001** | **0.002** |
| **Cold Spell Alt-3** | **≥3 days, T_min_ ≤ -6.0°C (P05)§** | **23 (1.2%)** | **1,563** | **67.96** | **1.117 (1.037-1.202)** | **0.004** | **0.009** |
| **OTHER METEOROLOGICAL VARIABLES** | | | | | | | |
| Low Solar Radiation | ≤ 109.0 J/cm² (Q25) | 472 (25.0%) | 31,841 | 67.46 | 1.006 (0.982-1.030) | 0.618 | 0.721 |
| High Solar Radiation | ≥ 281.4 J/cm² (Q75) | 472 (25.0%) | 26,500 | 56.14 | 1.004 (0.980-1.029) | 0.744 | 0.821 |
| Low Humidity | < 50% | 197 (10.4%) | 11,746 | 59.62 | 1.000 (0.966-1.034) | 0.981 | 0.981 |
| High Humidity | > 70% | 864 (45.8%) | 55,718 | 64.49 | 0.988 (0.965-1.012) | 0.333 | 0.462 |

Systematic evaluation of weather exposure definitions. Incidence rate ratios were estimated for primary extreme temperature exposures, mutually exclusive temperature categories, alternative heatwave and cold spell definitions, and other meteorological variables. Adjusted models used negative binomial regression with temporal fixed effects and cluster-robust standard errors. Bold entries indicate statistical significance after false discovery rate (FDR) correction.

Alternative exposure definitions:

- *Heatwave Alt-1:* ≥2 days with Tavg ≥27.1 °C (P95)
- *Heatwave Alt-2:* ≥2 days with Tavg ≥28.2 °C (P98)
- *Heatwave Alt-3:* ≥2 days with Tavg ≥28.8 °C (P99)
- *Cold Spell Alt-1:* ≥2 days with Tmin ≤-6.0 °C (P05)
- *Cold Spell Alt-2:* ≥2 days with Tmin ≤-12.1 °C (P01)
- *Cold Spell Alt-3:* ≥3 days with Tmin ≤-6.0 °C (P05)

Abbreviations: CI, confidence interval; FDR, false discovery rate; IRR, incidence rate ratio; OHCA, out-of-hospital cardiac arrest; P01/P02/P05/P95/P98/P99, temperature percentiles; Q25/Q75, quartiles; Tavg, daily average temperature; Tmax, daily maximum temperature; Tmin, daily minimum temperature.

**Table S3: Sex-Specific Incidence Rate Ratios for Primary Heatwaves, Cold Spells, and Severe Cold Days**

| **Temperature Exposure** | **Male IRR (95% CI)** | **Male p-value** | **Female IRR (95% CI)** | **Female p-value** | **Interaction p-value** |
| --- | --- | --- | --- | --- | --- |
| **Primary Heatwave** | 1.114 (1.024-1.212) | 0.013 | 1.203 (1.097-1.319) | <0.001 | 0.184 |
| **Primary Cold Spell** | 1.185 (1.068-1.315) | 0.002 | 1.194* | 0.003 | 0.892 |
| **Severe Cold Day** | 1.102 (0.963-1.261) | 0.160 | 1.201 (1.067-1.352) | 0.003 | 0.312 |
| **Global Interaction Test** |  |  |  |  | 0.226 |

Sex-specific incidence rate ratios for primary extreme temperature exposures.
Incidence rate ratios were estimated from sex-stratified negative binomial models using the same specification as the primary analysis. While effect estimates appeared slightly higher in women than in men, the global interaction test indicated no statistically significant sex modification of temperature-OHCA associations (p=0.226). Abbreviations: IRR, incidence rate ratio; CI, confidence interval; OHCA, out-of-hospital cardiac arrest.

**Table S4: Evaluation of Hungarian Meteorological Service Weather Alert Thresholds in Relation to OHCA Incidence**

| **HMS Alert Level** | **Definition** | **Alert Days, n (%)** | **OHCA Cases, n** | **IRR (95% CI)** | **p-value** |
| --- | --- | --- | --- | --- | --- |
| **HEAT-RELATED ALERTS** | | | | | |
| **Heatwave Level I** | **T_avg_ ≥ 25°C, 1 day** | **138 (7.3%)** | **7,321** | **1.059 (1.008-1.113)** | **0.024** |
| **Heatwave Level II** | **T_avg_ ≥ 25°C, 3 days OR T_avg_ ≥ 27°C, 1 day** | **89 (4.7%)** | **4,698** | **1.072 (1.027-1.119)** | **0.002** |
| Heatwave Level III | T_avg_ ≥ 27°C, 3 days | 20 (1.1%) | 1,098 | 1.140 (0.989-1.313) | 0.070 |
| **COLD-RELATED ALERTS** | | | | | |
| Frost Alert | T_min_ < 0°C | 324 (17.2%) | 21,186 | 0.972 (0.941-1.004) | 0.083 |
| **Severe Cold Alert** | **T_min_ < -10°C** | **41 (2.2%)** | **2,768** | **1.150 (1.019-1.297)** | **0.023** |
| Ice Alert | T_max_ < 0°C | 47 (2.5%) | 3,039 | 1.023 (0.947-1.104) | 0.567 |

Negative binomial added-effect models were applied using official HMS alert thresholds as binary exposures. Heatwave alerts showed a clear stepwise pattern: Level I was associated with a modest but significant increase, Level II yielded the most consistent and statistically robust effect, while Level III indicated the largest effect size but lacked precision due to rarity. Severe Cold Alerts (Tmin < -10 °C) produced estimates nearly identical to the primary severe cold day definition, providing strong confirmation of the consistency and clinical relevance of this threshold. Abbreviations: CI, confidence interval; HMS, Hungarian Meteorological Service; IRR, incidence rate ratio; OHCA, out-of-hospital cardiac arrest; T_avg_, daily average temperature; T_min_, daily minimum temperature; Tmax, daily maximum temperature.

**Table S5: Model Diagnostics and Sensitivity Analyses**

| **Analysis Type** | **Method/Specification** | **IRR (95% CI)** | **p-value** | **AIC** |
| --- | --- | --- | --- | --- |
| **PRIMARY ANALYSIS** | | | | |
| Baseline Model | Negative Binomial, cluster-robust SE | 1.143 (1.012-1.291) | 0.031 | 19,358.2 |
| **MODEL FAMILY COMPARISON** | | | | |
| Alternative Specification | Poisson with robust SE | 1.139 (1.012-1.282) | 0.030 | 19,683.1 |
| Overdispersion Test | NB dispersion parameter α | — | <0.001 | — |
| **TEMPORAL ROBUSTNESS (LEAVE-ONE-WINTER-OUT)** | | | | |
| Exclude Winter 2018/19 | 62 days excluded | 1.215 (1.067-1.383) | 0.003 | — |
| Exclude Winter 2019/20 | 91 days excluded | 1.138 (0.992-1.305) | 0.065 | — |
| Exclude Winter 2020/21 | 90 days excluded | 1.148 (1.007-1.309) | 0.040 | — |
| Exclude Winter 2021/22 | 90 days excluded | 1.089 (0.947-1.253) | 0.237 | — |
| Exclude Winter 2022/23 | 90 days excluded | 1.174 (1.025-1.346) | 0.021 | — |
| **CLUSTERING SENSITIVITY** | | | | |
| Year Clustering | SE clustered by year | 1.143 (1.006-1.298) | 0.040 | — |
| Month Clustering | SE clustered by month | 1.143 (1.015-1.287) | 0.027 | — |
| Year-Month Clustering | SE clustered by year-month | 1.143 (1.012-1.291) | 0.031 | — |
| **SEASONAL ADJUSTMENT SENSITIVITY** | | | | |
| Month Fixed Effects | Categorical month terms | 1.143 (1.012-1.291) | 0.031 | 19,358.2 |
| Month Splines | Natural cubic splines (6 df) | 1.221 (1.073-1.391) | 0.003 | 19,361.8 |
| Harmonic Terms | Sine/cosine seasonal terms | 0.968 (0.848-1.106) | 0.635 | 19,402.3 |

Negative binomial regression was consistently favored over Poisson models, with a substantial AIC improvement confirming the importance of accounting for overdispersion. Leave-one-winter-out analyses demonstrated that the primary severe cold day association (Tmin < -10 °C) remained significant in most seasonal exclusions. Results were robust across alternative clustering schemes, with Month and Year-Month clustering yielding nearly identical estimates, indicating that the choice of temporal clustering does not materially affect interpretation. Seasonal adjustment methods varied in impact, with categorical month fixed effects providing the most stable control. Abbreviations: AIC, Akaike Information Criterion; CI, confidence interval; df, degrees of freedom; IRR, incidence rate ratio; NB, negative binomial; OHCA, out-of-hospital cardiac arrest; SE, standard error; Tmin, daily minimum temperature.

**Figure S1: Heatwave Exposure Characteristics in the Study Period**

**
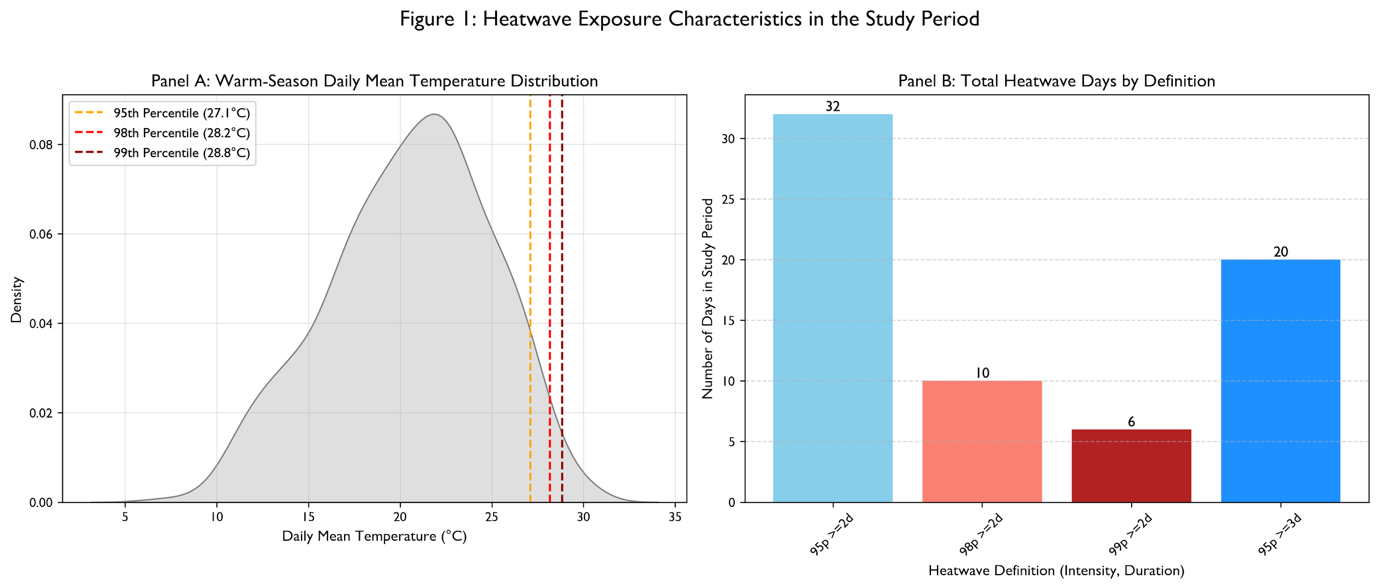
**

Panel A shows the distribution of daily average warm-season temperatures with vertical lines marking the 95th (27.1 °C), 98th (28.2 °C), and 99th (28.8 °C) percentiles used as intensity thresholds. Panel B displays the total number of exposure days identified for each heatwave definition across the 1,887-day study period, illustrating the progressive rarity of events as thresholds increase in intensity and duration. Abbreviations: T_avg_, daily average temperature.

**Figure S2: Cold Spell Exposure Characteristics in the Study Period**


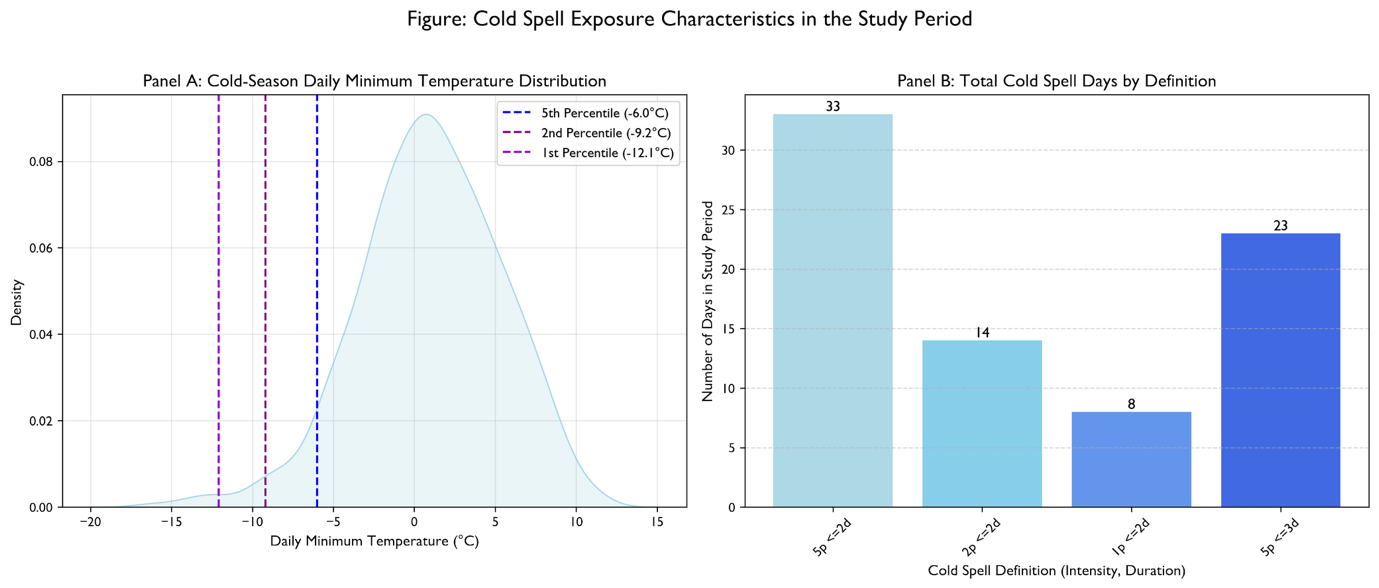


Panel A shows the distribution of daily minimum winter temperatures with vertical lines marking the 5th (-6.0 °C), 2nd (-9.2 °C), and 1st (-12.1 °C) percentiles used as thresholds. Panel B displays the total number of exposure days meeting alternative cold spell definitions during the study period, illustrating that more extreme thresholds correspond to progressively fewer events. Abbreviations: Tmin, daily minimum temperature.

**Figure S3: Added Effects of Cold Spells on OHCA Incidence**


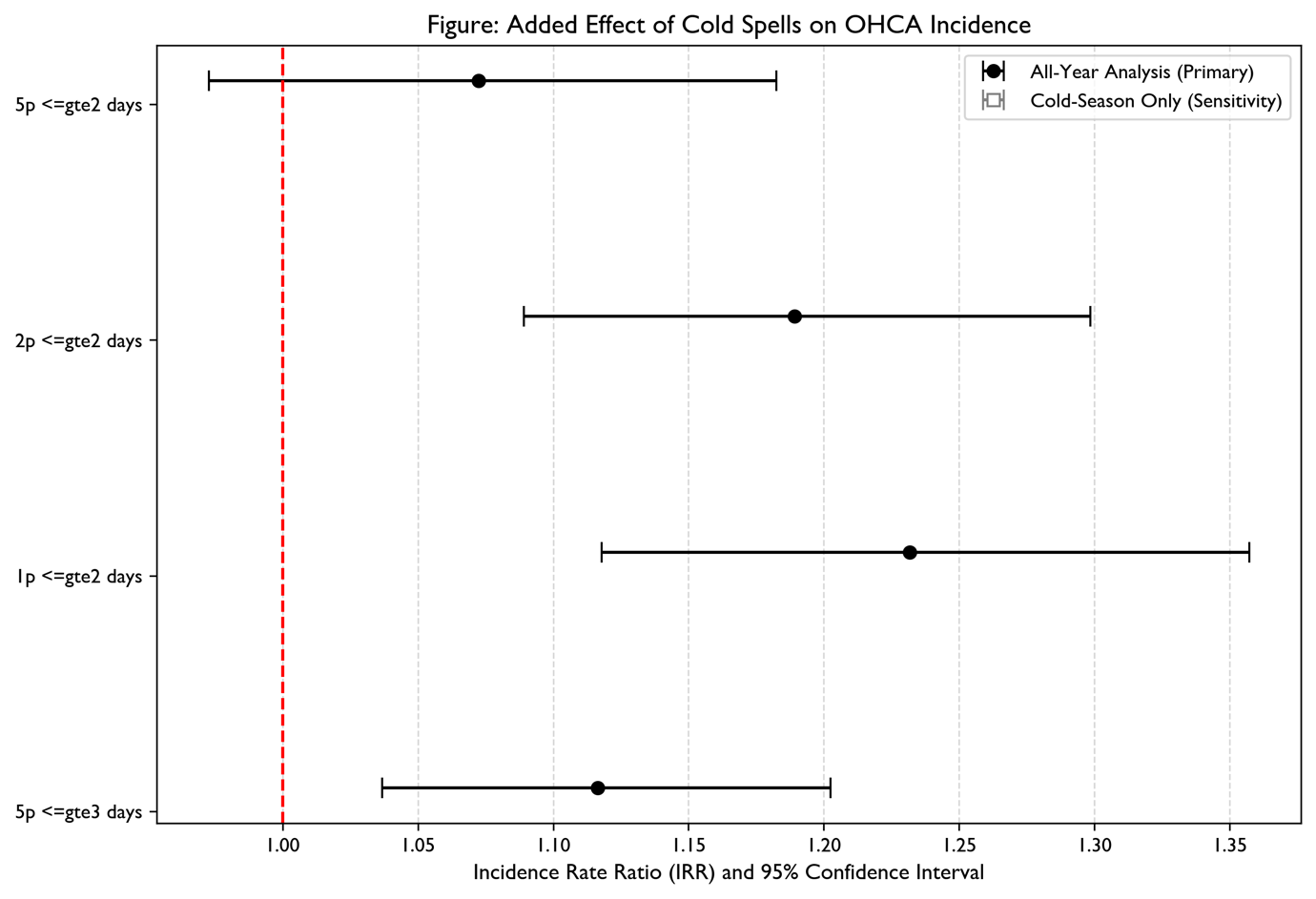

This forest plot shows results for alternative definitions of cold spells, based on different percentile thresholds of winter minimum temperature (5th, 2nd, and 1st percentiles) and duration requirements (≥2 or ≥3 consecutive days). The primary definition used in the main analysis (≥2 days at the 2nd percentile, -9.2 °C) is indicated. Colder and longer-lasting cold spells were generally associated with stronger increases in OHCA risk. Horizontal lines represent 95% confidence intervals. Abbreviations: CI, confidence interval; gte, “greater than or equal to”; IRR, incidence rate ratio; OHCA, out-of-hospital cardiac arrest; T_min_, daily minimum temperature.

**Figure S4: Stability of Primary Heatwave Finding (Leave-One-Summer-Out)**

**
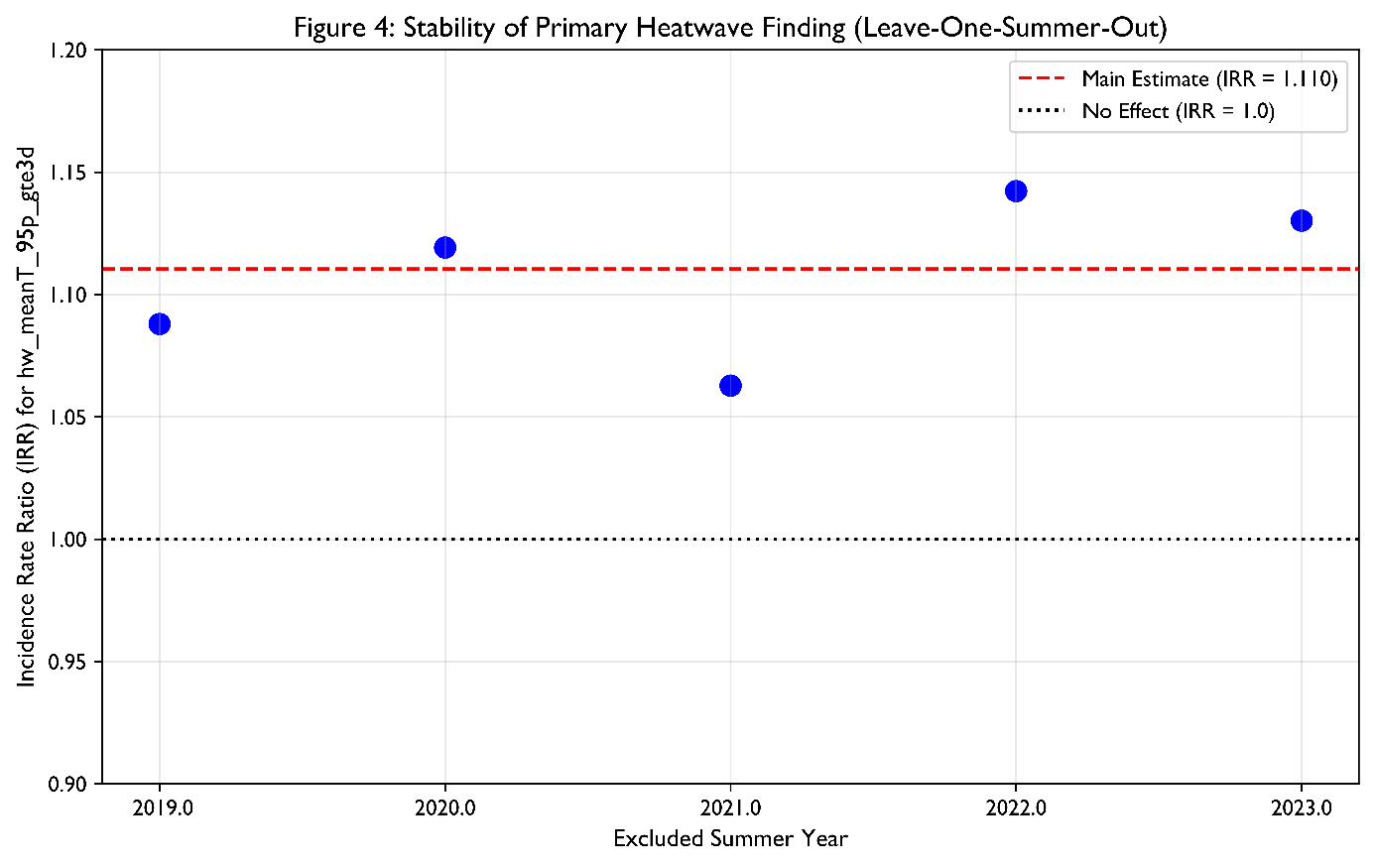
**

Each point shows the estimated IRR for heatwaves (≥3 days with Tavg ≥27.1°C) when one summer was omitted. The red dashed line marks the overall estimate from all years (IRR = 1.110). Abbreviations: gte, “greater than or equal to”; IRR, incidence rate ratio; LOSO, leave-one-season-out; Tavg, daily average temperature.

**Figure S5:** **Stability of Primary Cold Spell Finding (Leave-One-Winter-Out)**


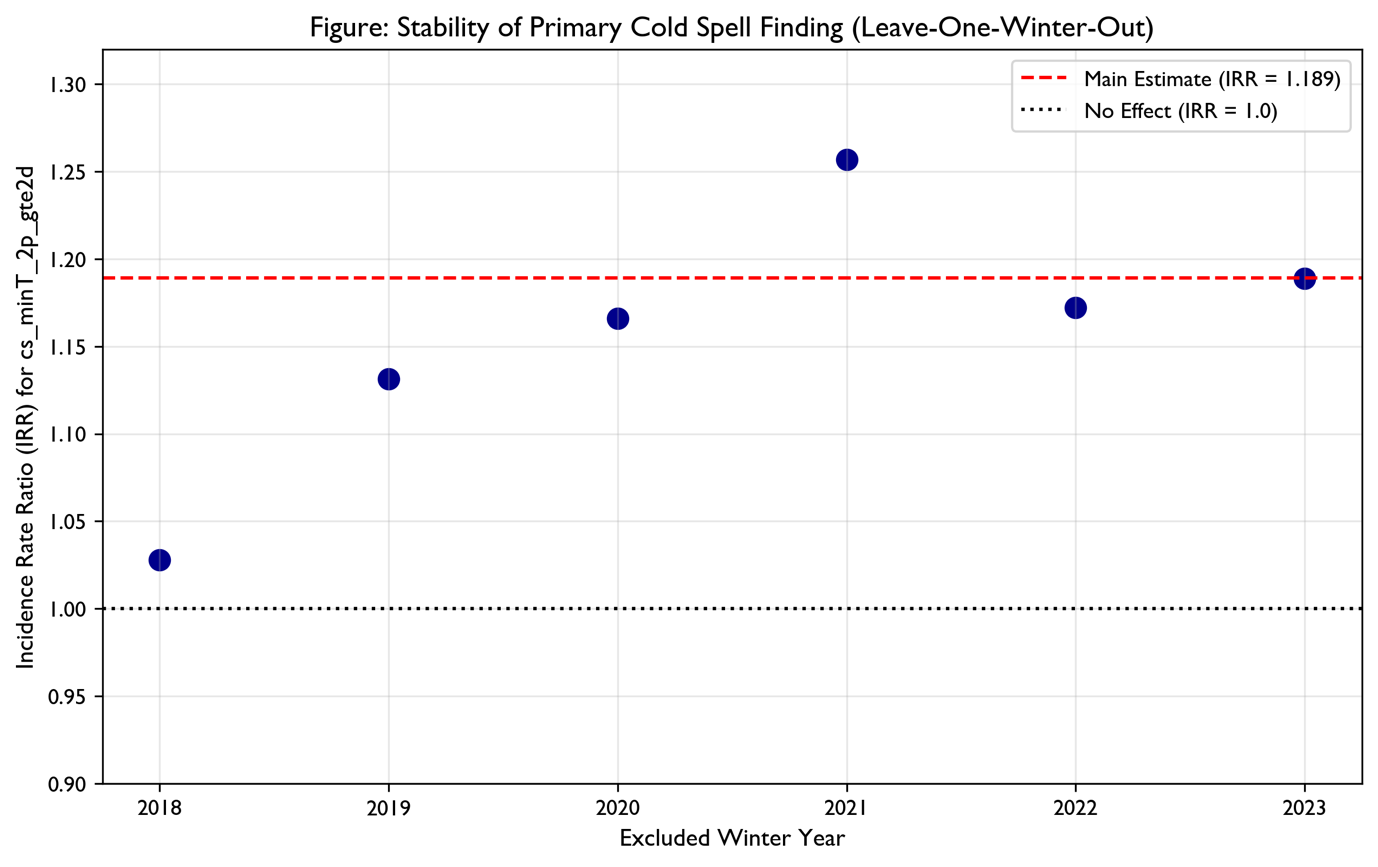

Each point shows the incidence rate ratio for cold spells (≥2 days with Tmin ≤ -9.2 °C) when one complete winter season (November-March) was excluded from the analysis. The red dashed line marks the overall estimate from all winters (IRR = 1.189). Results show that the association remained positive across most exclusions, indicating that no single winter period drives the overall effect. Horizontal lines represent 95% confidence intervals. Abbreviations: CI, confidence interval; IRR, incidence rate ratio; LOWO, leave-one-winter-out; T_min_, daily minimum temperature.

**Figure S6: Sex-Specific Analysis of Heatwave Effects on OHCA Incidence**


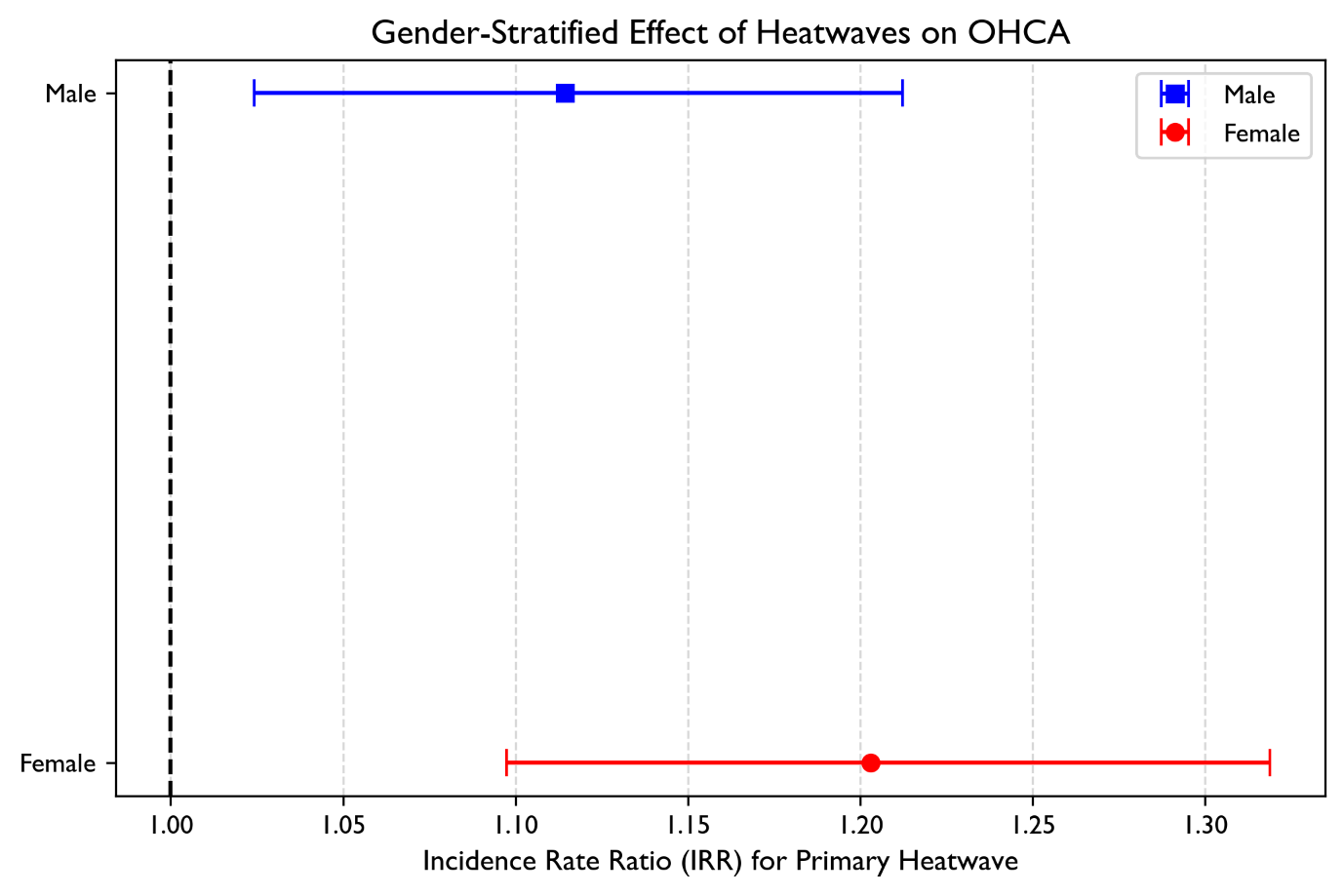


Forest plot showing incidence rate ratios for heatwaves (≥3 days with daily average temperature ≥27.1 °C) estimated separately for men and women using negative binomial models with identical specifications. Both sexes showed statistically significant increases in OHCA risk (male IRR 1.114, 95% CI: 1.024-1.212; female IRR 1.203, 95% CI: 1.097-1.319). Confidence intervals overlapped, and formal interaction testing indicated no statistically significant difference between sexes (p=0.184). Abbreviations: CI, confidence interval; IRR, incidence rate ratio; OHCA, out-of-hospital cardiac arrest.

**Figure S7: Sex-Specific Analysis of Cold Spell Effects on OHCA Incidence**

**
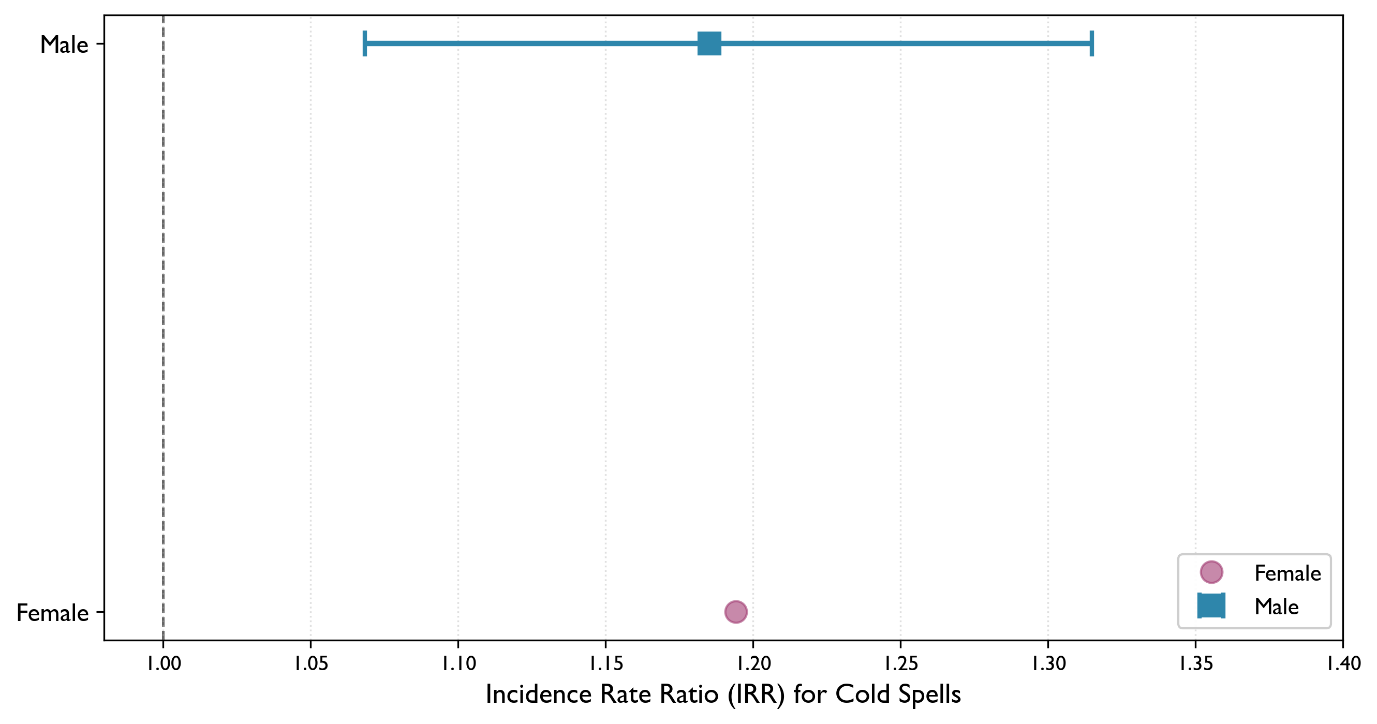
**

Forest plot showing incidence rate ratios for cold spells (≥2 days with daily minimum temperature ≤ -9.2 °C) estimated separately for men and women using negative binomial models with identical specifications. The male estimate showed a significant positive association (IRR 1.185, 95% CI: 1.068-1.315), comparable to the pooled analysis. The female model yielded an unstable estimate with an infinitely wide confidence interval, reflecting the rarity of cold spell events and limited case numbers. Abbreviations: CI, confidence interval; IRR, incidence rate ratio; OHCA, out-of-hospital cardiac arrest.

**Figure S8: Solar Radiation Effects on Daily OHCA Incidence**

**
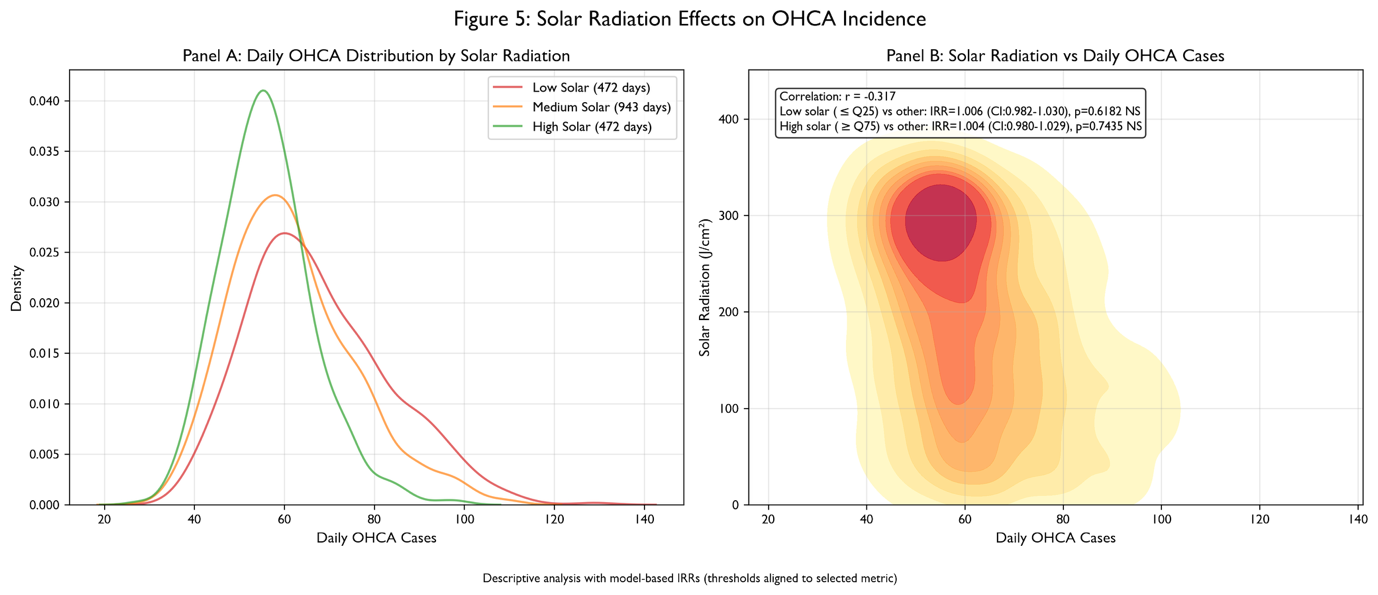
**

This figure examines the relationship between solar radiation and daily OHCA incidence. Panel A shows the distribution of OHCA cases across low, medium, and high solar radiation categories, with largely overlapping patterns. Panel B presents a density plot of OHCA cases by solar radiation levels, showing no consistent trend. Statistical analyses confirmed no significant associations for either low or high solar radiation. These findings support the specificity of temperature effects, indicating that OHCA risk is not significantly influenced by broader meteorological factors such as solar radiation. Abbreviations: CI, confidence interval; IRR, incidence rate ratio; OHCA, out-of-hospital cardiac arrest; Q, quartile.

**Figure S9: Humidity Effects on Daily OHCA Incidence**

**
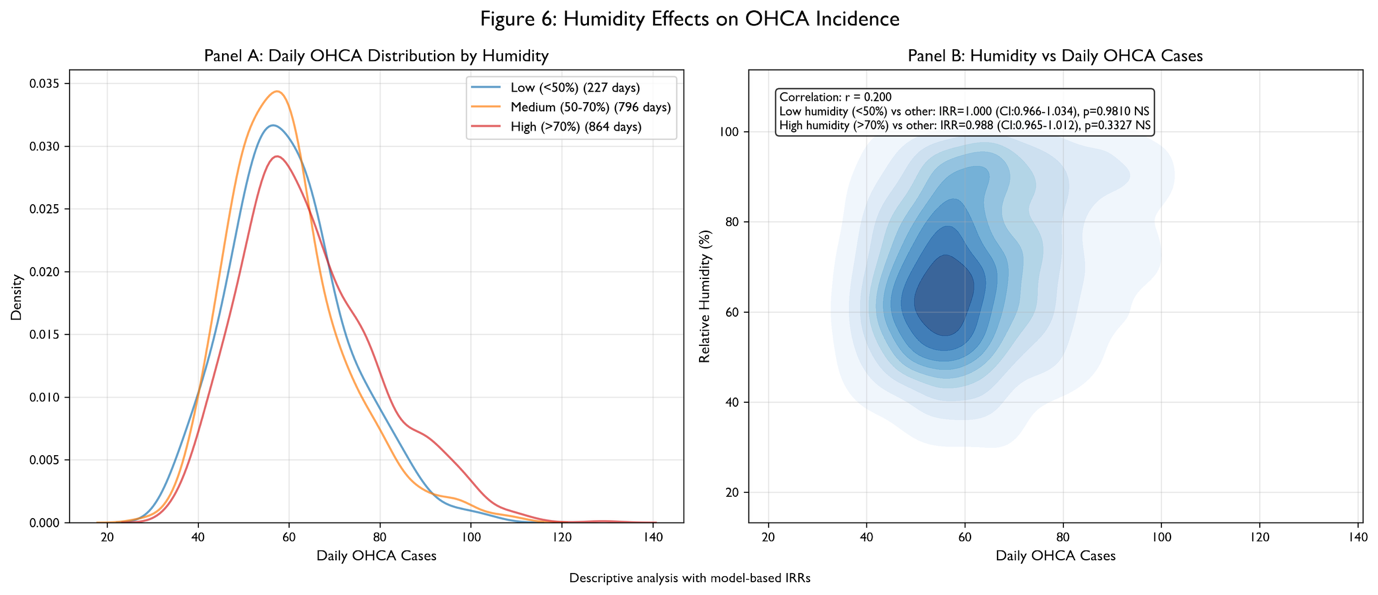
**

This figure shows the relationship between relative humidity and daily OHCA incidence. Panel A displays OHCA distributions across low (<50%), medium (50-70%), and high (>70%) humidity levels, which were highly overlapping. Panel B illustrates the joint distribution of OHCA cases and humidity, showing no consistent trend. Statistical models confirmed no significant associations for either low or high humidity categories. These findings support the specificity of temperature effects, indicating that humidity did not contribute to OHCA risk in this patient cohort. Abbreviations: CI, confidence interval; IRR, incidence rate ratio; OHCA, out-of-hospital cardiac arrest.
